# Mechanical Stability and Clinical Outcomes Following Posterior Cervical Fusion Surgery Using C3-6 Lateral Mass Screw Fixation: En Bloc Versus Regional Screw Fixation

**DOI:** 10.3390/jcm14041185

**Published:** 2025-02-11

**Authors:** Dong-Ho Lee, Sang Yun Seok, Woon Sang Lee, Hyung Rae Lee, Sehan Park, Chang Ju Hwang, Jae Hwan Cho

**Affiliations:** 1 Department of Orthopedic Surgery, Asan Medical Center, Seoul 05505, Republic of Korea; osdlee@gmail.com (D.-H.L.); guruws@naver.com (W.S.L.); birdone86@gmail.com (S.P.); basky47@gmail.com (C.J.H.); spinecjh@gmail.com (J.H.C.); 2 Department of Orthopedic Surgery, Daejeon Eulji Medical Center, Daejeon 35233, Republic of Korea; 3 Department of Orthopedic Surgery, Korea University Anam Hospital, Seoul 02841, Republic of Korea; drhrleeos@gmail.com

**Keywords:** en bloc fusion, mechanical failure, nonunion, posterior cervical fusion, regional fusion

## Abstract

**Background/Objectives**: Although lateral mass screws lower the risk of vertebral artery injury, they are shorter and have a weaker purchase than a pedicle screw, thereby posing the risk of mechanical failure following a ≥3-level posterior cervical fusion (PCF). Therefore, the purpose of this study is to demonstrate that the posterior en bloc fusion of C2-7 is mechanically stronger than shorter, regionally confined posterior fusions of the cervical spine. **Methods**: We included 178 patients who underwent PCF with ≥3 levels. Patients who underwent PCF that included both C2 and C7 were classified as the en bloc fusion group (EBF group, *n* = 116), while PCF cases not including these levels were assigned to a regional fusion group (RF group, *n* = 62). The fusion rate, incidences of mechanical failure, and clinical outcomes were evaluated using univariate analysis between the two groups. **Results**: The fusion rate was significantly higher in the EBF group than in the RF group (*p* = 0.038). In contrast, the mechanical failure rate was significantly lower in the EBF group (8/116 [6.9%] vs. 16/62 [25.8%], *p* = 0.047). Although the ROM was significantly higher in the RF group (*p* < 0.001), the functional scores did not significantly differ between the two groups. **Conclusions**: EBF seems to lower the rate of mechanical failure, as well as similar clinical outcomes, compared to RF. When the possibility of mechanical failure is high after PCF, extending the fusion level to C2 and C7 could be considered to minimize mechanical failure, rather than stopping at C3 or C6.

## 1. Introduction

Compression or ischemia of the cervical spinal cord due to degenerative cervical diseases causes a clinical syndrome known as cervical spondylotic myelopathy (CSM). This condition often presents non-specific symptoms, such as reduced balance, upper and lower extremity weakness, and urinary incontinence [1]. CSM is a common disorder that requires surgical treatment including anterior, posterior, and combined approaches to the cervical spine [2]. The anterior approach, including anterior cervical discectomy and fusion and artificial disc replacement, can be performed primarily for short-level decompression with good alignment with anterior main pathology [3]. In comparison, posterior cervical laminectomy and fusion (PCF) are mainly performed for multi-level decompression and the fusion or correction of kyphotic deformities. The use of this technique is increasing, and the results are encouraging [4,5].

It is notable, however, that Truumees et al. have reported that the incidence of pseudoarthrosis in PCF-treated patients is about 21.6% due to extensive muscle detachment [6]. In addition, during posterior fusion using only lateral mass screw fixation, mechanical stability may be lacking due to the difficulty in inserting long screws compared to posterior fusion using pedicle screws [7,8,9]. Accordingly, it is known that the probability of mechanical complications such as nonunion or screw failure is high during long-level posterior fusion ≥3 levels [5]. For this reason, some of these cases require revision surgery [10,11,12]. According to a study on subsidence in relation to the anterior approach, osteoporosis and polymethyl–methacrylate cages are risk factors, and the use of polyether–ether–ketone titanium cages and the preservation of the anterior edge lowers the risk of subsidence [3]. However, there are not many studies related to postoperative cervical stability following PCF.

Since the cervical spine has a larger range of motion (ROM) than the thoracolumbar spine, it is hypothesized that the en bloc fusion of C2-7 levels would be advantageous in reducing mechanical failure when performing long-level fusion ≥3 levels. In general, in the case of thoracolumbar fusion using only pedicle screws, mechanical failure may increase during long-level fusion, but unlike this, in the case of the cervical spine, when using the en bloc fusion of C2-7 levels, a C2 pedicle or pars screw and a C7 pedicle screw can be used to improve mechanical stability [4,5]. There have been no previous studies comparing mechanical stability and clinical outcomes between en bloc fusion including C2-7 and regional fixation in long-level PCF. Therefore, the purpose of our present study is to demonstrate that minimizing the fusion level in cases of long-level fusion ≥3 levels may cause mechanical failures such as metal failures and nonunion, especially since the en bloc fusion of levels C2-7 plays an important role in cervical stability.

## 2. Materials and Methods

### 2.1. Study Design

This study was approved by the institutional review board of our hospital, which waived the requirement for informed consent because of the retrospective nature of the data analysis (no. A20201303). We retrospectively reviewed the charts of 178 patients who underwent a PCF, for multi-level CSM (≥3 levels), between January 2011 and December 2022. All these cases were followed up for more than 2 years. The study protocol was approved by the institutional review board of our hospital, which waived the requirement for patient informed consent because of the retrospective nature of the data analysis.

**Figure 1:**
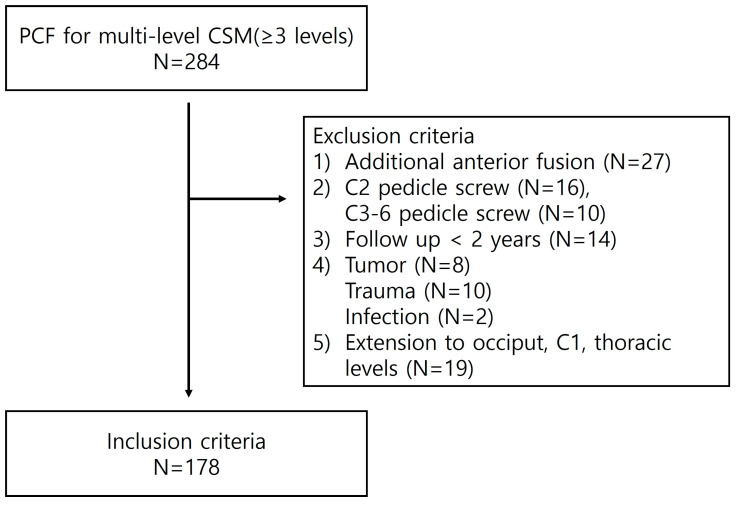
Flow chart of the study showing inclusion and exclusion criteria.

The study exclusion criteria were as follows: (1) additional anterior fusion surgery was performed simultaneously; (2) a screw fixation procedure was used other than the C2 pars screw, C3-6 lateral mass screw, or C7 pedicle screw technique; (3) the follow-up was less than 2 years; (4) other diseases were present, including tumors, traumas, and infections; or (5) if screw fixation was performed to extend the occiput, C1, or thoracic level (Figure 1). Patients were categorized based on the fusion level, i.e., a fusion that included both C2 and C7 (en bloc fusion [EBF] group, *n* = 116), or C2 and C7 were not included in the fusion segment (regional fusion [RF] group, *n* = 62).

### 2.2. Surgical Procedures and Postoperative Management

All surgical procedures for the study patients were performed by a single spine surgeon (D.H.L.). At the start of the surgery, the patient was placed in the prone position, under general anesthesia, with the head slightly flexed using Mayfield skeletal traction. This approach was then extended to the caudal 1/3 of C2 and cranial 1/3 of the C7 spinous process level to visualize the entry of the screw insertion. The lateral margins of the lateral masses were exposed at each level.

At the C2 level, after determining the pars screw insertion entry at 2–3 mm superior from the midline of the inferior articular process, a hole was made using a 2 mm diamond burr parallel to the C2-3 facet joint. At the C3 to C6 levels, a similar hole was made for lateral mass screw insertion (cephalad of 15°, diverging by 30°) using the technique of An and colleagues [13]. For the C7 pedicle screw, the entry point was made 1 mm laterally to the center of the lateral mass screw. The hole was made to converge according to the transverse axis of each pedicle. Each screw hole was then expanded using a drill bit through the burr hole, and bone bleeding was controlled with bone wax.

Prior to the laminectomy, a ligamentum flavectomy was performed at the upper and lower levels of the laminectomy site using pituitary forceps. The laminectomy was performed commencing at the lamina–facet junction.

Following the laminectomy, fixation was performed for each screw upon the pre-made hole. The length of the screw size was measured preoperatively using a computed tomography (CT) scan for as long as possible. After segmental screw fixation, appropriate neck extension was performed using Mayfield traction, and the cervical lordosis was then restored to ideal position. Curved rod-segmental screw fixation was then conducted. A local bone graft was performed using auto-bone (laminectomy auto-bone or iliac crest harvest bone) and demineralized bone matrix, followed by decortication of the lateral masses and the facet joints at the screw lateral region. The surgical approach was carefully performed to avoid injury to the semispinalis cervicis and nuchal ligament at the C2 and C7 levels, respectively. Any injury was repaired surgically. Following the surgery, all patients were encouraged to wear a rigid cervical neck collar for 8–12 weeks, and supervised rehabilitation was commenced for neck strengthening and stretching [14].

### 2.3. Radiological Analysis

#### 2.3.1. Evaluation of Fusion-Related Complications

Metal failure, non-union, and adjacent segment disease (ASD) were evaluated as fusion-related complications. Metal failure was assessed using the following criteria: (1) radiolucency around the screws (halo sign > 1 mm); (2) evidence of pull-out or change in the screw placement status as viewed on a CT scan 1 year after surgery; (3) fracture of a metal component, such as a screw or rod; or (4) disassembly of fixed constructs. Bone union was defined by the following criteria: (1) apparent bone continuity observed between the graft bone and posterior elements, such as the posterior arch, lamina, and facet joints, on lateral radiographs or a CT scan and (2) apparent lack of intervertebral mobility inside the fixation range observed on three lateral functional radiographs (extension, neutral, and flexion positions). If neither of these criteria was met, the fixation was defined as an apparent non-union. The diagnosis of ASD was defined as a case with symptoms including neck pain, radiating pain, and neurologic deficit related to radiographic changes among patients who had no symptoms for at least 6 months after previous fusion surgery.

#### 2.3.2. Evaluation of Cervical Alignment

**Figure 2:**
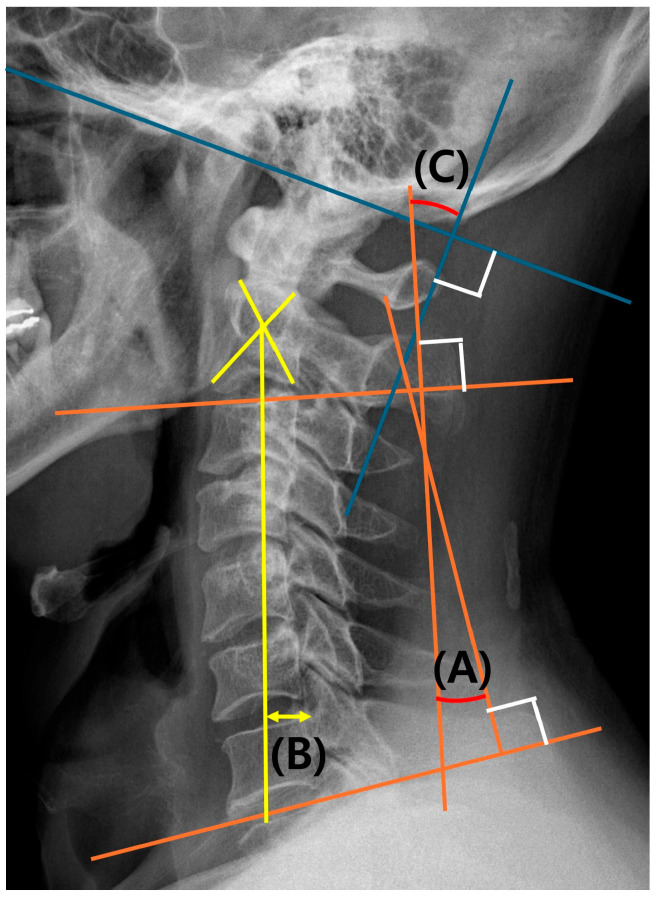
Radiologic parameters including C2-7 lordosis, C0-7 lordosis, and C2-7 sagittal vertical axis. (A) C2-7 lordosis; (B) C2-7 sagittal vertical axis (SVA); (C) C0-2 lordosis; (A + C) C0-7 lordosis.

C2-7 lordosis at the neutral position was evaluated preoperatively and at 2 years postoperatively using simple lateral radiographs. C2-7 lordosis was measured as the Cobb angle between the lower endplates of C2 and C7. The C2-7 sagittal vertical axis (SVA) was measured as the distance from the posterior–superior corner of C7 to a vertical line from the center of the C2 vertebra. The C0-2 lordosis is measured by the angle between the McGregor line and the low endplate of the C2 vertebra. The C0-7 lordosis is the sum of the values of C0-2 lordosis and C2-7 lordosis [15]. In addition, the difference between C0-7 lordosis at the flexion and extension position was defined as the ROM. The reason for using C0-7 lordosis for ROM measurements was to check residual ROM at C0-2 in case of C2-7 fixation. C0-7 lordosis was measured as the Cobb angle between the McGregor line and the line connecting the C7 lower endplate (Figure 2).

### 2.4. Clinical Outcomes and Functional Scores

Clinical outcomes and functional scores were measured using information from patient records on the preoperative and 2-year postoperative VAS for neck pain (VAS-N) and the Annex 1 Korean Neck Disability Index (AKNDI).

### 2.5. Statistical Analyses

The paired *t*-test, Chi-squared test, and Fisher’s exact test were used to compare the differences in each parameter between the study groups. All statistical analyses were performed using SPSS Statistics 21.0 (IBM, Armonk, NY, USA). *p*-values < 0.05 were considered significant.

## 3. Results

### 3.1. Demographics and Operative Factors

The mean age of the study patients at the index operation was 62.4 ± 12.6 years, and the mean follow-up period for this cohort was 42.2 ± 17.0 months (minimum follow-up was 2 years). The patient cohort compromised 100 (56.2%) cases of CSM due to ossification of the posterior longitudinal ligament and 78 (43.8%) cases due to spondylosis (with or without a herniated intervertebral disc, kyphotic deformity, or instability). The fusion ranges according to the number of vertebrae that had been fixed were as follows: four vertebrae (three levels) in 34 (19.1%) patients, five in 28 (15.7%) patients, and six in 116 (65.2%) patients. The decompression levels were 4.2 ± 0.5 in the EBF group and 3.6 ± 0.4 in the RF group (*p* < 0.001). The fusion levels were 6.0 in the EBF group and 4.4 ± 0.5 in the RF group (*p* < 0.001). The other data for these cases are summarized in Table 1.

### 3.2. Radiological Factors

#### 3.2.1. Fusion-Related Complications

**Figure 3:**
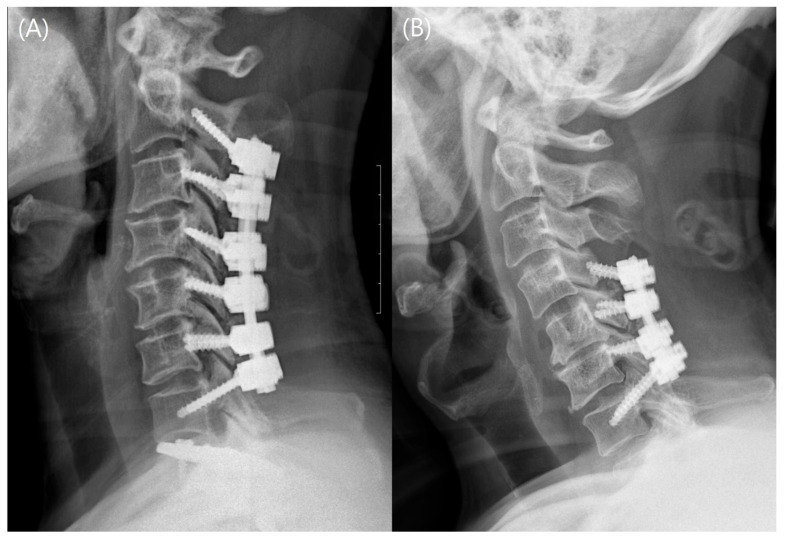
Representative cases. (**A**) Postoperative 1-year cervical lateral radiograph of an EBF patient. No apparent screw failure was observed on follow-up radiograph. (**B**) Postoperative 1-year cervical lateral radiograph of an RF patient. The proximal screw failure is confirmed on follow-up radiograph.

The fusion rate was significantly higher in the EBF group than in the RF group on radiograph and CT (*p* = 0.038 and 0.038, respectively). Eighteen patients (29.0%) in the RF group experienced various complications, namely, mechanical failure [screw breakage (two patients, [3.2%]; one distally), screw loosening (fourteen patients, [22.6%]; eight proximal and six distal], and ASD (two patients, [3.2%])]. In contrast, in the EBF group, four patients had a broken screw (3.4%, four distally), four had screw loosening (3.4%; four proximally), and six had ASD (5.2%). Representative cases are shown in Figure 3.

#### 3.2.2. Cervical Alignment

C2-7 lordosis and C2-C7 SVA did not significantly differ between the groups at the final follow-up (*p* = 0.553 and 0.324, respectively), although the cervical ROM was significantly higher in the RF group (*p* < 0.001; Table 2).

### 3.3. Clinical Outcomes and Functional Scores

Improvements in the VAS in terms of neck and arm pain and the AKNDI scores at each follow-up did not significantly differ between the groups. The number of reoperations with symptomatic complications was two in the EBF group and four in the RF group (Table 3).

## 4. Discussion

Surgeons agree on the requirement for an operation in patients with severe myelopathy symptoms or cord compression, but there is no consensus on how the range of lesion involvement differs according to a patient’s sagittal alignment or on which surgical method should be used in any given case [1]. In the case of multi-segment surgery, it is technically demanding to perform an operation using the anterior approach [2]. In addition, multi-level anterior decompression is known to be associated with a higher risk of complications, such as pseudarthrosis, implant failure, neurologic aggravation, and so on [16,17,18]. Surgeons thus tend to prefer a posterior approach in cases of a multi-level decompression of more than three segments.

However, it has been reported that long-segment PCF has a risk of implant failure, which increases the likelihood of reoperation [10,11,12]. Nagashima et al. reported implant failure in 25 of 51 patients (49%) who underwent multi-level PCF at 6 months postoperative CT scans [10]. According to Fayed et al., a revision operation was required in 15 (10.1%) of 149 patients who underwent PCF, of which 7 (4.7%) were construct-related procedures with mean follow-ups of 18.9 months [11]. Hines et al. reported a reoperation rate of 4.8% in a retrospective study of 369 cases of long-segment PCF with 2 years follow-up [12]. Similarly, we found in our present study that multi-level PCF increased the incidence of mechanical failure (22/89, 24.7%), and there was a reoperation rate of 3.3% in our patient series.

During PCF, lateral mass screws have proven to be relatively safe despite their proximity to the vertebral arteries, cervical nerve roots, and spinal cord but have inherent biomechanical limitations because of the small amount of bony purchase available in these areas [19,20,21]. Therefore, the most common complications associated with the PCF technique using lateral mass screw fixation are screw loosening or breakage, particularly toward the upper and lower extremes of the cervical spine, where the lateral masses are typically diminutive, and the pull-out resistance is low [6,7,8,9]. Since mechanical stability is not sufficient with only the lateral mass screw fixations in long-level fixation, C2 and C7 screws can be additionally used through en bloc fusion. Additionally, C2 pars screw and C7 pedicle screw fixations have also been reported to be easier and safer in prior investigations. Punyarat et al. reported that only 1 of 219 C2 pars screws in a series they evaluated caused occipital neuralgia due to superior breach, even with the free-hand technique [21]. A C7 pedicle screw is also safer than pedicle screw fixation of the subaxial cervical spine when considering the risk of vertebral artery injury. Clifton et al. described a free-hand pedicle screw placement success rate of 90% (36/40) and only four minor breaches (grade 1) were identified on CT in their study cohort, without neurologic complications [22].

We further found from our analyses that EBF may have a lower ROM than RF, but our data indicated no significant differences in terms of patient discomfort. Park et al. reported that 25 cases that underwent C2-7 posterior screw fusion maintained an average ROM of 30.4 ± 10.6° [23]. When fused from the occiput to below T1, motion was nearly nonexistent. However, the patients in whom the O-C2 was left unfused maintained substantial ROM.

Here, we conducted a comparative study on C2-7 fusion and on multiple fusions of less than C2-7. Nagashiwa et al. recommended in their prior study not to stop caudally at the C7 level but to additionally include the thoracic level or use a C6 pedicle screw for firm fixation [7]. However, we have reported previously in another study that ending the fusion at C7 (even if C7 is the caudal fixation level, it can be replaced) did not cause adverse impacts in terms of C7-T1 segment failure, the fusion rate, neck pain, or neurologic outcomes [24]. This study included only patients who were fixed from C2 to C7, excluding occipital, C1, and upper thoracic levels, and confirmed that en bloc fixation could provide sufficient mechanical stability.

There were some potential limitations in our present study of note. First, its retrospective design may have induced a patient selection bias. Second, the quality of the evidence was limited because of the small sample size derived from our single institution only. Third, selection bias in surgical methods (decompression level, each screw’s mean size, and bone graft material) may have existed, but no statistically significant difference was observed between the two groups. Fourth, no additional research has been conducted on which patients should consider EBF over RF. For example, in patients with osteoporosis, it would be advantageous to perform EBF compared to RF because the risk of mechanical failure is high. However, since a subgroup analysis was not conducted on this, an additional follow-up study is needed. Lastly, the fusion level and fixation method were not homogenous within the RF group. However, this study completely excluded the occipital, C1, and upper thoracic levels, which can mainly affect the ROM and mechanical stability, from the fixation level. Additionally, the screws used were also only pars screws for C2, pedicle screws for C7, and lateral mass screws for C3-6. If the regional screw fixation group could be analyzed by subgroup analysis, the reliability of this study could be increased. However, if the regional screw fixation group is divided by fixation levels, it can be divided into six subgroups, as confirmed in Table 1, and the sample size of each group will be too small to analyze in one institution, so additional research such as a multi-center study should be followed. Despite these limitations, our current findings on mechanical stability after PCF may be meaningful, as they provide additional information that can assist with preoperative planning for these surgeries.

## 5. Conclusions

If a cervical fusion of level 3 or higher is required, screw-related complications might be reduced when EBF is performed and includes both the C2 and C7 levels, with no differences in clinical outcomes when compared to RF. The use of the EBF technique to obtain firm fixation and a cervical fusion of level 3 or higher may thus potentially yield better results.

## Figures and Tables

**Table 1:** Comparison of demographic and operative factors between two groups.

Parameter	EBF Group	RF Group	*p*-Value
	(*n* = 116)	(*n* = 62)	
Age	63.7 ± 12.4	60.0 ± 12.8	0.192
Sex			1.000
Male	72 (62.1%)	40 (64.5%)	
Female	44 (37.9%)	22 (35.5%)	
BMI	25.4 ± 4.4	25.8 ± 4.1	0.721
BMD (spine)	0.68 ± 0.38	0.78 ± 0.35	0.254
Smoking	16 (13.7%)	12 (19.3%)	0.547
HTN	48 (41.4%)	22 (35.5%)	0.317
DM	18 (15.5%)	12 (19.4%)	0.192
Malignancy	4 (3.4%)	0 (0.0%)	0.713
Decompression levels	4.2 ± 0.5	3.6 ± 0.4	<0.001 *
Fusion levels	6.0	4.4 ± 0.5	<0.001 *
- 4 levels (C2-3-4-5)	0	4 (6.5)	
- 4 levels (C3-4-5-6)	0	27 (43.5)	
- 4 levels (C4-5-6-7)	0	3 (4.8)	
- 5 levels (C2-3-4-5-6)	0	9 (14.5)	
- 5 levels (C3-4-5-6-7)	0	19 (30.7)	
- 6 levels (C2-3-4-5-6-7)	116 (100.0)	0	
Mean lateral mass screw length	13.8 ± 1.1	14.3 ± 1.1	0.097
Mean pedicle screw length	26.2 ± 3.1	27.2 ± 2.2	0.854
Iliac crest bone graft	34 (29.3%)	12 (19.4%)	0.725
Follow-up (m)	42.5 ± 16.6	41.6 ± 18.0	0.812

EBF, en bloc fusion; RF, regional fusion; BMI, body mass index; BMD, bone mineral density; HTN, hypertension; DM, diabetes mellitus; m, months. * *p* < 0.05.

**Table 2:** Comparison of radiologic factors between the two groups.

			EBF Group *n* = 116	RF Group *n* = 62	*p*-Value
Preoperative	Lordosis (C2-C7)	Degree	−9.7 ± 11.1	−13.2 ± 11.1	0.171
	Range of motion	Degree	49.4 ± 15.6	52.8 ± 16.7	0.363
	C2-C7 SVA	mm	19.7 ± 18.0	24.3 ± 19.5	0.137
Postoperative, 1 year	Lordosis (C2-C7)	Degree	−16.9 ± 8.5	−12.3 ± 10.5	0.034 *
		Change	−7.2 ± 13.3	0.9 ± 10.1	<0.001 *
		*p*-value	<0.001 *	0.634	
	Range of motion	Degree	30.6 ± 13.7	43.1 ± 16.1	<0.001
		Change	−18.9 ± 14.6	−9.8 ± 16.5	0.001
		*p*-value	<0.001 *	<0.001 *	
	C2-C7 SVA	mm	25.5 ± 15.7	24.3 ± 13.2	0.724
		Change	5.7 ± 10.9	−0.1 ± 15.7	0.047 *
		*p*-value	<0.001 *	0.915	
Final follow-up	Lordosis (C2-C7)	Degree	−14.2 ± 10.4	−12.8 ± 10.3	0.553
		Change	−4.3 ± 13.9	0.4 ± 12.2	0.115
		*p*-value	0.027 *	0.863	
	Range of motion	Degree	27.1 ± 13.3	40.8 ± 15.8	<0.001 *
		Change	−22.1 ± 14.7	−12.3 ± 16.9	<0.001 *
		*p*-value	<0.001 *	<0.001 *	
	C2-C7 SVA	mm	27.7 ± 19.3	23.7 ± 14.1	0.324
		Change	7.9 ± 16.5	−0.6 ± 6.3	0.027 *
		*p*-value	<0.001 *	0.627	
Fusion, complications		Fusion (radiograph)	110 (94.8%)	48 (77.4%)	0.038 *
		Fusion (CT)	110 (94.8%)	48 (77.4%)	0.038 *
		Mechanical failure	8 (6.9%)	16 (25.8%)	0.047 *
		- Screw loosening	4 (3.4%)	14 (22.6%)	<0.001 *
		- Screw broken	4 (3.4%)	2 (3.2%)	1.000
		ASD	6 (5.1%)	2 (3.2%)	1.000

EBF, en bloc fusion; RF, regional fusion; y, years; SVA, sagittal vertical axis; CT, computed tomography; ASD, adjacent segment degeneration. * *p* < 0.05. * The *p*-value that can be seen on the horizontal side is a statistical analysis of the difference between the two groups. The *p*-value that can be confirmed on the vertical side is a statistical analysis of the change value 1 year after surgery and the final follow-up period value compared to the preoperative value.

**Table 3:** The comparison of clinical scores between two groups.

			EBF Group *n* = 116	RF Group *n* = 62	*p*-Value
Preoperative	Neck pain	VAS score	4.0 ± 2.7	4.8 ± 3.0	0.187
	Arm pain	VAS score	4.7 ± 2.9	4.4 ± 2.7	0.633
	NDI	Score	18.8 ± 7.5	19.6 ± 7.7	0.644
Postoperative, 1 year	Neck pain	VAS score	2.4 ± 2.2	3.2 ± 2.6	0.152
		Change	−1.6 ± 2.7	−1.7 ± 3.5	0.891
		*p*-value	<0.001 *	<0.001 *	
	Arm pain	VAS score	3.1 ± 2.7	3.1 ± 2.8	0.993
		Change	−1.5 ± 3.1	−1.3 ± 3.2	0.688
		*p*-value	<0.001 *	<0.001 *	
	NDI	Score	13.3 ± 7.3	13.5 ± 7.6	0.914
		Change	−5.5 ± 6.1	−6.0 ± 7.3	0.736
		*p*-value	<0.001 *	<0.001 *	
Final follow-up	Neck pain	VAS score	2.6 ± 2.1	2.8 ± 2.4	0.595
		Change	−1.5 ± 2.0	−2.0 ± 3.3	0.435
		*p*-value	<0.001 *	<0.001 *	
	Arm pain	VAS score	3.2 ± 2.6	3.1 ± 2.3	0.846
		Change	−1.4 ± 3.7	−1.3 ± 3.1	0.813
		*p*-value	<0.001 *	<0.001 *	
	NDI	Score	12.4 ± 6.2	12.9 ± 7.4	0.717
		Change	−6.4 ± 8.0	−6.6 ± 7.4	0.893
		*p*-value	<0.001 *	<0.001 *	

EBF, en bloc fusion; RF, regional fusion; NDI, neck disability index; VAS, Visual Analog Scale; y, years. * *p* < 0.05. * The *p*-value that can be seen on the horizontal side is a statistical analysis of the difference between the two groups. The *p*-value that can be confirmed on the vertical side is a statistical analysis of the change value 1 year after surgery and the final follow-up period value compared to the preoperative value.

## Data Availability

The data presented in this study are available upon request from the corresponding author.
